# Association between workplace violence and occupational stress among emergency department nurses: a cross-sectional study

**DOI:** 10.3389/fpubh.2025.1603651

**Published:** 2025-08-07

**Authors:** Lixi Long, Nan Xie, Xiaoli Chen, Hao Zhang, Luying Zhong, Dongmei Diao, Ling Zhu, Yue Zhou

**Affiliations:** ^1^Department of Emergency Medicine, West China Hospital, Sichuan University/West China School of Nursing, Sichuan University, Chengdu, China; ^2^Disaster Medical Center, Sichuan University, Chengdu, China; ^3^Nursing Key Laboratory of Sichuan Province, Chengdu, China

**Keywords:** emergency department nurses, workplace violence, occupational stress, association, a cross-sectional study

## Abstract

**Background:**

Emergency department (ED) nurses suffer from workplace violence (WPV) and occupational stress (OS) due to the working environment. However, a relatively small number of studies on the relationship between WPV and OS among ED nurses have been conducted and its impact on nurse health or nursing quality.

**Methods:**

A cross-sectional study was conducted in 30 hospitals in China from December 26, 2023, to January 18, 2024 through questionnaire survey and stratified cluster sampling.

**Results:**

A total of 1,540 ED nurses were surveyed, 1,309 of whom had experienced WPV. OS score of these ED nurses was (55.55 ± 16.78). Correlation between WPV and OS was significant (rs = 0.577, *P* < 0.01), and multivariate regression analysis revealed that an education level of bachelor's degree or higher; weekly working hours of 41–48, 49–58, and ≥59 h; physical violence experience; and psychological WPV were key influencing factors of OS among ED nurses.

**Conclusion:**

A high proportion of ED nurses had experienced WPV, they had moderate to severe levels of OS, and WPV was a direct predictor of OS among them. This may be reduced by strengthening the management of violence in their workplace.

## 1 Introduction

Workplace violence (WPV) is a common phenomenon in healthcare. The World Health Organization defines WPV as: “incidents where staff are abused, threatened, or assaulted in circumstances related to their work, involving an explicit or implicit challenge to their safety, well being or health (1).” Moreover, between 19.3 and 36.4% of hospital workers have experienced WPV, and 66.9% have experienced non-physical violence (2, 3). Forty-one studies involving 42,222 nurses from 13 countries revealed that the overall incidences of WPV, verbal abuse, physical violence, threatening behavior, physical assault, sexual harassment, and bullying/mobbing were 58%, 64%, 23%, 30%, 21%, 12%, and 25%, respectively (4). Owing to the high-stress and high-risk work environment in emergency departments (ED), ED nurses are one of the groups that are at the highest risk for WPV in hospitals. A multicenter study in Taiwan revealed that 92.9% of ED nurses had experienced WPV in the past 2 years (5). In Italy, 91.5% of ED nurses have experienced verbal or physical violence (6). In Saudi Arabia, 73.7% of ED nurses experienced violence in the past 2 years, with 47.4% having experienced physical violence and 94.3% having experienced non-physical violence (7). Furthermore, in Turkey, 96.5% of ED nurses reported experiencing verbal violence (8). In China, WPV affects the sleep health of ED nurses (9). WPV not only reduces the motivation in nurses but also adversely affects them at physical, psychological, social, and professional levels; moreover, it is one of the main reasons for nurse turnover (10). Additionally, WPV can lead to compassion fatigue in healthcare workers, which in turn reduces professional efficacy (11–13).

Occupational stress (OS), also known as work stress, is a physiological and psychological reaction when occupational demands exceed an individual's abilities (14). According to the World Health Organization, stress can be defined as a state of worry or mental tension caused by a difficult situation. Stress is a natural human response that prompts us to address challenges and threats in our lives. Everyone experiences stress to some degree. The way we respond to stress, however, makes a big difference to our overall well being (15). Karasek and Theorell's (16) Job Demands-Control (JDC) Model argues that adverse health-related outcomes, both psychological and physiological, arise from a combination of high job demand and a low level of job control. Johnson and Hall (17) expanded the JDC model as the JDC-Support (JDCS) model by introducing the social support. The JDCS model emphasizes that organizations or leaders will give tangible and intangible resources based on the working situation of employees, and assist employees to effectively control the operation of work tasks and their psychological stress. When job stressors are not consistently relieved with available resources such as social support, negative consequences likely occur, including sickness absenteeism, and job turnover. The transactional model of stress and coping, proposed by Folkman and Lazarus (18), conceptualizes stress as a highly individualized phenomenon arising from person-environment interactions. This model posits that both available resources and perceived stressors significantly influence the resulting stress response, which in turn serves as a robust predictor of depression. OS has been recognized as one of the major occupational health hazards affecting practitioners globally, and it seriously endangers their physical and mental health (19). The International Labor Organization reports that OS has become the second most reported work-related health issue in European countries, indicating that OS has become an important factor affecting the health of the working population (20). Studies have shown that OS has a significant positive effect on depression in the working population, i.e., the higher the OS level in the working population, the greater the likelihood of the occurrence of depressive symptoms (21). A study revealed that 65.3% of Chinese workers experience OS (22–25).

Workers in the medical field typically face more stressful environments than those in other industries. In China, a study reported that 26.5% of the 1,077 nursing participants experienced effort–reward imbalance (ERI) (26). Furthermore, a questionnaire sent to 2,689 nurses in all regions of Germany showed that nurses put in more effort than their reward (ERI ratio of 1.7), 38.3% of the nurses considered leaving their jobs several times a month or had left their jobs multiple times, and 30.6% were considering changing employers (27). In Iran, 81% of nurses suffer from occupational stress (28). Chronic stress: this occurs when the stressor persists over an extended period. Prolonged exposure to chronic stress can lead to cumulative physiological and psychological effects, increasing the risk of health problems such as cardiovascular disease, anxiety, and depression (29). Chronic stress activates the hypothalamus-pituitary-adrenal (HPA) axis and sympathetic-adrenal-medullary axis, which in turn secrete glucocorticoids and catecholamines. These hormones may temporarily suppress inflammation, in turn, it reduces the body's adverse health reactions, such as reducing the risk of depression, diabetes, cardiovascular disease, and cancer. However, the lasting stress exposure induce HPA “fatigue,” glucocorticoid-resistance, and nuclear factor kappa-B activation, which in turn promote proinflammatory cytokines, ultimately causing inflammation that may induce various diseases (30). Mental health problems at work (e.g., OS, burnout, or depressive tendencies) have become one of the highly studied topics of interest and research in the field of occupational health in China and other countries in recent years. The heavy workload of healthcare workers in ED, who primarily treat critically ill patients, has led to more pronounced OS for doctors and nurses in this department (31, 32). Moreover, a cross-sectional study of 17,582 ED nurses in China revealed direct and indirect mediating effects of ERI on the intention to resign (33). WPV has a seriously negative impact on nurses, patients, and healthcare systems, and OS is a major challenge to modern occupational health and safety. A study by Rasool et al. (34) found that WPV significantly increased OS in healthcare workers. However, a relatively small number of studies on the relationship between WPV and OS among ED nurses have been conducted. Through a cross-sectional survey, this study aimed to understand the current situation of WPV and OS among ED nurses and explore their relationship to provide a scientific basis for improving the work environment and alleviating OS for ED nurses.

## 2 Design and methods

### 2.1 Aim

To explore the correlation between WPV and OS among ED nurses, and elucidate the effect of WPV on OS among ED nurses.

### 2.2 Study design and setting

This was a cross-sectional survey study, based on the previous research of the team, the minimum sample size required 1,330 cases (35, 36), a total of 1,540 ED nurses were included.

### 2.3 Study subjects

#### 2.3.1 Inclusion and exclusion criteria

Inclusion criteria: (1) licensed nurses in the ED; and (2) ≥1 year of working experience in emergency medicine.

Exclusion criteria: (1) nurses with a history of mental illness; (2) nurses on maternity leave or breastfeeding (≥1 month); and (3) nurses in training.

#### 2.3.2 Sampling method

Stratified cluster sampling was used in this study. Based on the geographic regions of China (east, central, south, north, southwest, northwest, and northeast), with the support from the Chinese Nursing Association, 2–3 provincial capital cities in each stratum and 2–3 tertiary hospitals in each city were sampled, and ED nursing workers who were eligible (aged ≥18 years, no history of mental illness, had not taken psychotropic medications in the week prior to the survey) were enrolled in the study.

### 2.4 Research instruments

The survey included general sociological information, a survey on WPV and working conditions, and the health status of nurses.

General baseline information: includes age, sex, education, years of service, night shifts, marital status, parity, income, etc.

WPV: a WPV questionnaire adapted by Havaei et al. (37) from Statistics Canada's 2005 National Survey of the Work and Health of Nurses (38) and Hesketh et al.'s questionnaire (39) were used to investigate emergency nurses' exposure to WPV in the preceding year, consisting of the following four items: (a) emotional abuse from patients and/or their family, (b) emotional abuse from employers and/or coworkers, (c) physical abuse by patients and/or their family, and (d) physical abuse by employers and/or coworkers at their primary workplace in the past year (0 = never, 6 = daily). Mean scores (0–12) were obtained, with higher scores indicating more frequent WPV experiences. The severity of WPV was determined based on the mean score: normal was <1, mild was 1–2, moderate was 2–3, and severe was ≥3. Higher average scores indicated more frequent exposure to WPV. The Cronbach's alpha coefficient was 0.71.

Occupational stress questionnaire (OSQ): a questionnaire developed by Professor Johannes Siegrist (40) of Germany to evaluate OS factors based on the ERI model. The questionnaire has been shown to reasonably measure and compare work-related stress in psychometric tests based on sociological theories by comparative analysis of data from five European countries. The questionnaire consisted of six items on effort, 11 items on reward, and five items on over-commitment. Each item in the questionnaire was scored on a 5-point Likert scale of 1–5. A higher score indicated a higher level of OS. The Cronbach's alpha coefficient was 0.82.

### 2.5 Data collection

Wenjuanxing was used to generate the electronic version of the survey questionnaire. One individual was assigned as the core person-in-charge in each hospital surveyed who distributed the QR code link and collected and explained the survey to the participants. Furthermore, informed consent was obtained from all participants. All the questions were set as mandatory to ensure complete response, and each participant was allowed to answer only once to avoid repeated answers.

### 2.6 Statistical analysis methods

The SPSS 26.0 statistical package was used for data analysis. Frequency and percentage were used to describe count data, and mean and standard deviation were used to describe normally distributed measurement data; independent samples *t*-test or one-way analysis of variance was used to compare the OS scores of different characteristics, and the correlation between the score of each WPV domain (physical violence, psychological violence, and the total score of violence) and the score of each OS domain (effort, reward, over-commitment, and the total score of OS) was analyzed using Pearson's correlation test; stratified regression was used to analyze the factors affecting OS.

## 3 Results

### 3.1 Descriptive analysis of general demographic information of the study subjects

In this study, 1,550 questionnaires were collected, 10 questionnaires with all answers being the same were excluded. In total, 1,540 questionnaires were included for analysis. Among the respondents, 1,211 (78.6%) were women, 980 (63.6%) were married, 1,354 (87.9%) had a bachelor's degree or higher, 716 (46.5%) had a monthly income of ≥10,000 yuan, and 907 (58.9%) held a junior title. A total of 1,309 participants or 85.0% had experienced WPV (Table 1).

**Table 1:** Sociodemographic characteristics (*n* = 1,540).

**Variables**	**Categories**	**Number**	**Frequency (%)**
Age	20–29 years	582	37.8
	30–39 years	734	47.7
	40–49 years	183	11.9
	≥50 years	41	2.6
Gender	Female	1,211	78.6
	Male	329	21.4
Marital status	Single	560	36.4
	Married	980	63.6
Fertility status	No	710	46.1
	Yes	830	53.9
Education level	Junior college	186	12.1
	Bachelor's degree or higher	1,354	87.9
Professional titles	Senior nurse	907	58.9
	Nurses in charge	574	37.3
	Co-chief nurse and above	59	3.8
Number of years spent working	≤ 5 years	491	31.9
	6–10 years	493	32.0
	11–15 years	300	19.5
	16–20 years	121	7.8
	>20 years	135	8.8
Working hours per week	<40 h per week	577	37.5
	41–48 h per week	780	50.6
	49–58 h per week	123	8.0
	≥59 h per week	60	3.9
Night shift	Yes	1,359	88.2
	None	181	11.8
Number of night shift	None	181	11.8
	1–4 times per month	239	15.5
	5–8 times per month	659	42.8
	≥9 times per month	461	29.9
Monthly income (yuan)	<4,000 yuan	57	3.7
	4,000–55,999 yuan	149	9.7
	6,000–77,999 yuan	250	16.2
	8,000–99,999 yuan	368	23.9
	≥10,000 yuan	716	46.5
Workplace violence	Physical violence	943	61.3
	Psychological violence	1,299	84.4
	Total	1,309	85

### 3.2 Descriptive analysis of the results of the survey on WPV and OS

This study revealed that workplace physical violence, psychological violence, and total scores were (0.76 ± 0.98), (1.44 ± 1.17), and (2.20 ± 1.97), respectively, and effort, reward, over-commitment, and total scores for OS were (19.22 ± 5.35), (23.72 ± 9.10), (12.60 ± 4.74), and (55.55 ± 16.78), respectively (Table 2).

**Table 2:** Analysis of workplace violence and occupational stress scores.

**Variable**	**Dimensional**	**Min**	**Max**	**Mean**	**SD**
Workplace violence	Physical violence	0	6	0.76	0.98
	Psychological violence	0	6	1.44	1.17
	Total scores	0	12	2.20	1.97
Occupational stress	Effort	6	30	19.22	5.35
	Reward	11	55	23.72	9.10
	Over-commitment	5	25	12.60	4.74
	Total scores	22	110	55.55	16.78

### 3.3 Two-way correlation analysis of WPV and OS scores and subdomains

Correlation analysis showed that the total WPV score of the ED nurses was positively correlated with the total OS score, the total score of WPV was positively correlated with the effort, reward, and over-commitment scores under OS (rs = 0.408, 0.566, and 0.497, *P* < 0.01), and the total OS score was positively correlated with the physical violence and psychological violence scores under WPV (rs = 0.453, 0.592, *P* < 0.01; Table 3).

**Table 3:** Correlation coefficients among the study variables.

**Variable**			**Workplace violence**			**Occupational stres**			**The total OS score**
			**Physical violence**	**Psychological violence**	**The total violence score**	**Effort**	**Reward**	**Over-commitment**	
Workplace violence	Physical violence	rs	1						
		P							
	Psychological violence	rs	0.674^**^	1					
		P	0						
	The total violence score	rs	0.899^**^	0.929^**^	1				
		P	0	0					
Occupational stress	Effort	rs	0.264^**^	0.465^**^	0.408^**^	1			
		P	0	0	0				
	Reward	rs	0.482^**^	0.549^**^	0.566^**^	0.501^**^	1		
		P	0	0	0	0			
	Over-commitment	rs	0.382^**^	0.517^**^	0.497^**^	0.662^**^	0.759^**^	1	
		P	0	0	0	0	0		
	The total OS score	rs	0.453^**^	0.592^**^	0.577^**^	0.777^**^	0.916^**^	0.905^**^	1
		P	0	0	0	0	0	0	

^**^P <0.01.

## 4 Analysis of influencing factors of OS

### 4.1 Univariate analysis

Univariate analysis, with OS as the dependent variable and general demographic information and WPV as the independent variables, showed that the total OS score of the study subjects was 55.55 ± 16.78, and the differences were statistically significant (*P* < 0.05) between different age strata, marital status, parity, education, job titles, years of experience, weekly hours of work, and frequencies of night shifts (Table 4).

**Table 4:** Single-factor analysis of the occupational stress.

**Variable**	**Categories**	**Mean ±SD**	**t/F**	***P-*value**
Age	20–29 years	52.45 ± 17.40	11.674	<0.001
	30–39 years	57.41 ± 16.04		
	40–49 years	58.31 ± 16.29		
	≥50 years	53.76 ± 16.29		
Gender	Female	55.64 ± 16.33	0.418	0.676
	Male	55.18 ± 18.35		
Marital status	Single	53.12 ± 16.94	−4.307	<0.001
	Married	56.93 ± 16.54		
Fertility status	No	53.89 ± 16.93	−3.598	<0.001
	Yes	56.96 ± 16.52		
Education level	Junior college	49.68 ± 17.88	−5.128	<0.001
	Bachelor's degree or higher	56.35 ± 16.47		
Professional titles	Senior nurse	53.51 ± 16.94	19.955	<0.001
	Nurses in charge	57.92 ± 16.07		
	Co-chief nurse and above	63.76 ± 15.74		
Number of years spent working	≤ 5 years	51.97 ± 17.43	8.877	<0.001
	6–10 years	56.68 ± 16.39		
	11–15 years	57.31 ± 15.85		
	16–20 years	59.13 ± 16.36		
	>20 years	57.26 ± 16.21		
Working hours per week	<40 h per week	51.95 ± 15.52	24.032	<0.001
	41–48 h per week	56.37 ± 15.94		
	49–58 h per week	62.48 ± 19.24		
	≥59 h per week	65.15 ± 23.27		
Night shift	Yes	55.56 ± 16.87	0.107	0.915
	None	55.42 ± 16.12		
Number of night shift	None	55.42 ± 16.12	7.604	<0.001
	1–4 times per month	52.53 ± 14.57		
	5–8 times per month	54.70 ± 16.96		
	≥9 times per month	58.37 ± 17.47		
Monthly income	<4,000 yuan	58.61 ± 20.86	1.108	0.351
	4,000–55,999 yuan	52.45 ± 17.40		
	6,000–77,999 yuan	57.41 ± 16.04		
	8,000–99,999 yuan	58.31 ± 16.29		
	≥10,000 yuan	53.76 ± 16.29		

### 4.2 Multivariate analysis

Multivariate stratified analysis with OS score as the dependent variable showed that the correlation coefficients between OS and physical violence, psychological violence, and violence scores were 0.453, 0.592, and 0.577, respectively, which were all statistically significant, and all the variables were included in the multivariate linear regression for analysis. Collinearity diagnosis performed before stratified regression analysis revealed that the tolerances were all >0.1 and the variance inflation factors were all <10, indicating that there was no multicollinearity among the variables in this study (Table 5).

**Table 5:** Colinearity statistics.

**(Constant)**		**VIF**	**Allowance**
Age	20–29 years		
	30–39 years	0.218	4.592
	40–49 years	0.181	5.528
	≥50 years	0.359	2.784
Marital status	Single		
	Married	0.331	3.019
Fertility status	No		
	Yes	0.309	3.237
Education level	Junior college		
	Bachelor's degree or higher	0.915	1.093
Professional titles	Senior nurse		
	Nurses in charge	0.587	1.705
	Co-chief nurse and above	0.694	1.441
Number of years spent working	≤ 5 years		
	6–10 years	0.262	3.810
	11–15 years	0.235	4.258
	16–20 years	0.294	3.400
	>20 years	0.169	5.929
Working hours per week	<40 h per week		
	41–48 h per week	0.848	1.179
	49–58 h per week	0.859	1.165
	≥59 h per week	0.909	1.100
Number of night shift	None		
	1–4 times per month	0.436	2.291
	5–8 times per month	0.294	3.405
	≥9 times per month	0.316	3.160
Workplace violence	Physical violence	0.535	1.869
	Psychological violence	0.531	1.884

The multiple linear regression showed that an educational level of bachelor's degree or higher, weekly working hours of 41–48, 49–58, and ≥59 h, physical violence score, and psychological violence score were the influencing factors of OS score among ED nurses. The regression formula was established as OS = 3.055 × bachelor's degree or above (1) + 3.215 × weekly working hours 41–48 h (1)/4.825 × 49–58 h (1)/7.752 × ≥59 h (1)+ 1.480 × physical violence score + 7.160 × psychological violence score, i.e., the OS score increased by 3.055 for a subject with bachelor's degree or higher; the OS score increased by 7.16 (*P* < 0.05) for every increase of 1 point in the psychological violence score; the OS score increased by 3.215 (*P* < 0.05) for a weekly working duration of 41–48 h; the OS score increased by 4.825 (*P* < 0.05) for a weekly working duration of 49–58 h; the OS score increased by 7.752 (*P* < 0.05) for a weekly working duration of ≥59 h; and the OS score increased by 1.48 (*P* < 0.05) for every increase of 1 point in the physical violence score (Supplementary Table S1). The model used the adjusted *R*^2^ = 0.379, suggesting that the model explained 37.9% of the OS score.

## 5 Discussion

This study analyzed the current status of WPV and OS among ED nurses and explored the correlation between the two. Results of the study showed that ED nurses experienced a high rate of WPV and significant level of OS, and that WPV was a significant predictor of OS. This finding provides a new perspective for understanding occupational health risks among ED nurses.

ED nurses are at high risk for WPV due to the unique nature of their work environment. This study showed that 84.4% of ED nurses had experienced psychological violence, 61.3% had experienced physical violence, and the overall incidence of WPV was 85.0%. This number is higher than Kenya's statistics of 73.2% and Saudi Arabia's statistics of 73.7%, but lower than Taiwan's statistics of 92.9% (5, 7, 41). The differences may have stemmed from differences in the research instruments, sampling method, and regional culture, and may also be related to the hospital environment, policies and regulations, and level of training of healthcare professionals. Specifically, the high number of patients in the ED, the long waiting time, the complexity of patient conditions, and the anxiety of patients and their families all can lead to violence. Additionally, poor nurse–patient communication and inadequate capacity to respond to violence further exacerbate the risk of WPV.

OS is an important factor affecting the occupational health of nurses. This study showed that ED nurses had a high OS score (55.55 ± 16.78), which is consistent with previous results (42–44). However, Mirzaei et al. (45) and Rakhshani et al. (46) reported lower levels of OS in nurses, possibly due to the fact that patients in the ED are critically ill with rapidly changing conditions, and it is often difficult for the nurses to make accurate predictions regarding the changes, which leads to constant worry among the nurses, thus increasing their OS. Furthermore, compared with other nurses, ED nurses need to possess flexibility and adaptability to make quick responses in critical situations, which also increases their OS.

The analyses in this study found that the total WPV score was positively correlated with the total OS score, the total violence score for WPV was positively correlated with the effort, reward, and over-commitment scores for OS (rs = 0.408, 0.566, and 0.497, respectively, *P* < 0.01), and the total OS score was positively correlated with the physical violence and psychological violence scores for WPV (rs = 0.453, 0.592, respectively, *P* < 0.01), and different demographic characteristics had a significant effect on the OS level. Multivariate linear regression showed that ED nurses with a bachelor's degree or above had a higher level of OS, which may be attributed to the fact that this group had to undertake complex clinical tasks, teaching and management duties at the same time, resulting in higher stress (44, 47), and longer the weekly working hours of ED nurses led to higher level of OS, indicating that excessive workload and irregular work schedule and rest had a negative impact on the physical and mental health of nurses (43). The present study further found that physical and psychological violence was significant predictors of OS, especially psychological violence, as every 1-point increase in psychological violence led to an increase in the OS score by 7.16. This result supports the evidence from previous studies of the negative impact of WPV on mental health, i.e., (48–50), the more frequent the exposure to WPV, the higher the level of OS. Violence not only damages the physical health of nurses, but can also lead to mental problems such as anxiety, depression, and compassion fatigue, and these mental states may further reduce work efficacy and increase burnout and turnover intention (51, 52). Additionally, effort and over-commitment in OS were significantly associated with depressive symptoms, and lack of reward was a protective factor. Negative emotions may be exacerbated when nurses are in a chronic state of high effort and low reward, which poses a serious threat to mental health. Chronic psychological stress is correlated with serious consequences for individual health, the urgent need for concrete and systemic protective measures for medical personnel exposed to violence and chronic stress: (1) environmental modifications to optimize workplace safety, (2) systemic reforms to prevent and mitigate workplace violence, and (3) focus on individual and family needs and provide the necessary support.

## 6 Limitations

This study was the first to address the relationship between WPV and OS among ED nurses, and the findings provide a reference for other countries and groups of healthcare workers. However, this study still has a few limitations. First, the cross-sectional study design limited causality inference. Second, only ED nurses participated and only their data were used for analysis in this study, thus, the applicability of the findings in other departments needs to be further examined. Therefore, a longitudinal design can be used in future studies for detailed exploration of the effects of WPV on OS.

## 7 Conclusion

According to the present study, the incidence of WPV is high among ED nurses, with 85.0% of ED nurses having experienced WPV. WPV directly predicts OS; psychological violence is an important factor for OS; and WPV increases OS among ED nurses. These indicate the importance of improving the work environment, decreasing WPV, reducing the level of OS, and improving occupational health. These findings not only provide a theoretical basis for policy makers, but also suggest interventions for practitioners that can help improve the quality of life and quality of healthcare services of ED nurses.

## Data Availability

The raw data supporting the conclusions of this article will be made available by the authors, without undue reservation.
