# Supplementary material for: Association between workplace violence and occupational stress among emergency department nurses: a cross-sectional study
Source: Front Public Health. 2025 Aug 7;13:1603651. doi: 10.3389/fpubh.2025.1603651 (PMC12367734; doi:10.3389/fpubh.2025.1603651)
Supplement: Supplementary file 1 [file Data_Sheet_1.pdf]

Appendix 1.Results of multivariate linear regression analysis of occupational stress

Variables		Unstandardized coefficients		Standardized coefficients	t	*P*	95%CI	
		B	SE	Beta			Lower	Upper
(Constant)		37.599	1.613		23.305	＜0.001	34.435	40.764
Age	20-29 years							
	30-39 years	1.956	1.446	0.058	1.353	0.176	-0.880	4.793
	40-49 years	0.732	2.449	0.014	0.299	0.765	-4.071	5.536
	⩾50 years	-2.939	3.494	-0.028	-0.841	0.400	-9.792	3.915
Marital status	Single							
	Married	0.412	1.218	0.012	0.338	0.735	-1.976	2.800
Fertility status	No							
	Yes	-0.046	1.217	-0.001	-0.038	0.970	-2.432	2.340
Education level	Junior college							
	Bachelor’s degree or higher	3.055	1.081	0.059	2.826	0.005	0.934	5.176
Professional titles	Senior nurse							
	Nurses in charge	1.172	0.910	0.034	1.287	0.198	-0.614	2.957
	Co-chief nurse and above	3.749	2.108	0.043	1.779	0.075	-0.385	7.883
Number of years spent working	⩽5 years							
	6-10 years	-0.126	1.410	-0.004	-0.090	0.929	-2.892	2.640
	11-15 years	-1.332	1.756	-0.031	-0.759	0.448	-4.777	2.112
	16-20 years	0.318	2.310	0.005	0.138	0.891	-4.213	4.849
	＞20 years	3.208	2.902	0.054	1.105	0.269	-2.485	8.900
Working hours per week	＜40 h per week							

	41-48 h per week	3.215	0.732	0.096	4.392	＜0.001	1.779	4.651
	49-58 h per week	4.825	1.342	0.078	3.596	＜0.001	2.193	7.457
	⩾59 h per week	7.752	1.827	0.089	4.243	＜0.001	4.168	11.336
Number of night shift	None							
	1-4 times per month	-1.474	1.409	-0.032	-1.046	0.296	-4.238	1.290
	5-8 times per month	-0.345	1.257	-0.010	-0.275	0.784	-2.811	2.120
	⩾9 times per month	0.401	1.308	0.011	0.306	0.760	-2.166	2.967
Workplace violence	Physical violence	1.480	0.469	0.087	3.154	0.002	0.560	2.400
	Psychological violence	7.160	0.397	0.497	18.023	＜0.001	6.381	7.939
