# Supplementary material for: Association between workplace violence and occupational stress among emergency department nurses: a cross-sectional study
Source: Front Public Health. 2025 Aug 7;13:1603651. doi: 10.3389/fpubh.2025.1603651 (PMC12367734; doi:10.3389/fpubh.2025.1603651)
Supplement: Supplementary file 2 [file Data_Sheet_2.pdf]

Appendix 2 List of abbreviations

ED: Emergency department

WPV: workplace violence

OS: occupational stress

JDC: Job Demands-Control

JDCS: JDC-Support

ERI: effort–reward imbalance

HPA: hypothalamus-pituitary-adrenal

SAM: sympathetic-adrenal-medullary

GCs: glucocorticoids

CAs: catecholamines

NF-κB: nuclear factor kappa-B

OSQ: Occupational stress questionnaire

VIFs: variance inflation factors
